# Comparative Transcriptome Analysis of Male and Female Conelets and Development of Microsatellite Markers in *Pinus bungeana*, an Endemic Conifer in China

**DOI:** 10.3390/genes8120393

**Published:** 2017-12-19

**Authors:** Dong Duan, Yun Jia, Jie Yang, Zhong-Hu Li

**Affiliations:** Key Laboratory of Resource Biology and Biotechnology in Western China, Ministry of Education, College of Life Sciences, Northwest University, Xi’an 710069, China; Dongduan@nwu.edu.cn (D.D.); jy878683@163.com (Y.J.); jieyang@nwu.edu.cn (J.Y.)

**Keywords:** *Pinus bungeana*, sexual recognition, population structure, SSR marker, transcriptome

## Abstract

The sex determination in gymnosperms is still poorly characterized due to the lack of genomic/transcriptome resources and useful molecular genetic markers. To enhance our understanding of the molecular mechanisms of the determination of sexual recognition of reproductive structures in conifers, the transcriptome of male and female conelets were characterized in a Chinese endemic conifer species, *Pinus bungeana* Zucc. ex Endl. The 39.62 Gb high-throughput sequencing reads were obtained from two kinds of sexual conelets. After de novo assembly of the obtained reads, 85,305 unigenes were identified, 53,944 (63.23%) of which were annotated with public databases. A total of 12,073 differentially expressed genes were detected between the two types of sexes in *P. bungeana*, and 5766 (47.76%) of them were up-regulated in females. The Kyoto Encyclopedia of Genes and Genomes (KEGG) enriched analysis suggested that some of the genes were significantly associated with the sex determination process of *P. bungeana*, such as those involved in tryptophan metabolism, zeatin biosynthesis, and cysteine and methionine metabolism, and the phenylpropanoid biosynthesis pathways. Meanwhile, some important plant hormone pathways (e.g., the gibberellin (GA) pathway, carotenoid biosynthesis, and brassinosteroid biosynthesis (BR) pathway) that affected sexual determination were also induced in *P. bungeana*. In addition, 8791 expressed sequence tag-simple sequence repeats (EST-SSRs) from 7859 unigenes were detected in *P. bungeana*. The most abundant repeat types were dinucleotides (1926), followed by trinucleotides (1711). The dominant classes of the sequence repeat were A/T (4942) in mononucleotides and AT/AT (1283) in dinucleotides. Among these EST-SSRs, 84 pairs of primers were randomly selected for the characterization of potential molecular genetic markers. Finally, 19 polymorphic EST-SSR primers were characterized. We found low to moderate levels of genetic diversity (*N*_A_ = 1.754; *H*_O_ = 0.206; *H*_E_ = 0.205) across natural populations of *P. bungeana*. The cluster analysis revealed two distinct genetic groups for the six populations that were sampled in this endemic species, which might be caused by the fragmentation of habitats and long-term geographic isolation among different populations. Taken together, this work provides important insights into the molecular mechanisms of sexual identity in the reproductive organs of *P. bungeana*. The molecular genetic resources that were identified in this study will also facilitate further studies in functional genomics and population genetics in the *Pinus* species.

## 1. Introduction

In plants, totipotent meristematic cells usually experience a long development period, and then undergo the reproductive stage to form flowers, which are complex sexual organs [1]. The sexual dimorphism of plants (including hermaphrodite, monoecious, and dioecious, etc.) is related to morphological and physiological characteristics that differentiate male and female plant reproductive organs [2,3]. Generally, differential gene expression is considered as an important factor for sexually dichotomous phenotypes. The development and maintenance of sex-specific phenotypes are under a series of metabolic pathways and regulatory genetic networks where various connected sex differences in expression genes, transcription factors (TFs), and other regulators are associated [4,5]. Based on the recently detected genomic/transcriptome information resources, the morphological differences between the sexes are considered to be largely affected by the sex differences in the gene regulatory and expression pattern [6,7]. Some studies have suggested that the determinants of sex differentiation in plants (i.e., *Salix suchowensis* and cucumber) are significantly involved in the expression of sex chromosomes and sex determination genes [8,9]. Some other studies have found that plant hormones signal transduction (e.g., *ACS*, *ASR1*, *IAA2*, and *AUX* gene networks) also affected the gender differentiation and plant development process [10]. Recently, the transcriptome analysis for the complete flowers of cucumber showed that the genes participating in sexual differentiation were significantly related to the ethylene synthesis, carotenoid, and auxin biosynthesis pathways [8]. In addition, the downstream metabolic pathways and genetic networks that are essential for sex differentiation in plants may be controlled by upstream sex-determining genes [4]. However, up to now, many studies of the determinants of sexual identity have mainly focused on the model angiosperms species [7,11], and the molecular genetic mechanisms of sex recognition of gymnosperms are largely unclear.

In general, the gymnosperms have long generation times, large effective population sizes, and complex genomes, and these characteristics have hindered the accurate investigation and characterization of the sex differentiation genes at a genomics level [6,12]. In recent years, with the advance of next generation sequencing technology, transcriptome sequencing has proven to be an efficient and rapid method for determining the expression of different sexes. For example, expression pattern analysis has suggested that the *TERMINAL FLOWER 1* (*TFL1*)-like genes played an important role in the development process of reproductive organs and sex determinations of gymnosperms [13]. In addition, comparative transcriptome analysis of the two *Pinus tabuliformis* sexes indicated the occurrence of a sex-biased expression pattern in gymnosperms [7,11]. However, some other metabolism pathways that are affecting sex differentiation in gymnosperms still remain unknown.

*Pinus bungeana* Zucc. ex Endl., belonging to Sect. *Parrya* Mayr, is an economically and ecologically important soft conifer species, with a key role in local forest ecosystems. This species is mainly distributed in the mountain areas in Shaanxi, Shanxi, and Henan Provinces with an altitude of 500–1800 m, and is an endemic conifer in China. It is one of the main conifer species that have adapted to calcareous loess and mild saline soil in coniferous species. In addition, *P. bungeana* has strong resistance to sulfur dioxide and soot pollution in nature. In recent years, due to the over-cutting and fragmentations of natural habitats, the wild resources of *P. bungeana* populations have increasingly declined. Meanwhile, *P. bungeana* is outcrossing, anemophilous, and has different development positions of male and female conelets. These characteristics make this tree species an excellent model for studying differential gene expression and sexual recognition between the two sexes in conifer species.

Most previous studies on *P. bungeana* have mainly focused on its phylogenetic position [14,15,16], physiological ecology, and phylogeographic structure [17]. Meanwhile, some researchers have also investigated the genomic/transcriptome information and population genetics of *P. bungeana* [15,17]. On the other hand, the development and application of molecular genetic information is fundamental for the conservation of the wild species. In recent years, transcriptome sequencing provides a rapid and powerful tool for the development of molecular genetic tools, such as co-dominant simple sequence repeats (SSR) markers [8]. These transcriptome-based markers have been widely used to research phylogenetic evolution and species conservation of organisms [18,19].

In this study, we characterized the transcriptomes of male and female conelets of *P. bungeana* using Illumina high-throughput sequencing technology. This study was designed to enrich the molecular genetic resources for *P. bungeana*, to identify new candidate genes that are involved in sex determination and differentiation, and to detail the different expression patterns between the sexes. Furthermore, differentially expressed genes (DEGs) that are involved in metabolisms were further determined and analyzed. In addition, a set of novel expressed sequence tag-simple sequence repeats (EST-SSR) markers were developed from the transcriptome data. To effectively manage the wild resources of *P. bungeana*, the population genetic diversity and structure were also evaluated for the six sampled populations across most ranges of its natural distributions, using these new developed EST-SSR markers.

## 2. Materials and Methods

### 2.1. Plant Materials

*Pinus bungeana* is a monoecious conifer species, which produces male and female cones during March–May. Three male and three female conelets (female conelet 1, female conelet 2, female conelet 3, male conelet 1, male conelet 2, and male conelet 3) were separately collected from three normal trees at the campus of Northwest University, China in April 2013. We selected expanded (but un-pollinated conelets), that were frozen in liquid nitrogen for RNA isolation. In addition, we sampled fresh needles from six natural populations (Table 1, Figure S1) across most of the distributional ranges of *P. bungeana* to investigate the polymorphism of microsatellite primers and population structure.

### 2.2. RNA Isolation and Quality Assay

The TRIzol Reagent (Invitrogen, Carlsbad, CA, USA) was used to extract the total RNA of the samples, according to the manufacturer’s instructions. The quantity and quality of RNA were assessed by 1% gel electrophoresis and NanoPhotometer^®^ spectrophotometer (IMPLEN, Westlake Village, CA, USA). The RNA integrity was accurately assessed by the RNA Nano 6000 Assay Kit of the Agilent Bioanalyzer 2100 system (Agilent Technologies, Santa Clara, CA, USA).

### 2.3. Transcriptome Sequencing

Individual isolated RNA was used to construct cDNA libraries for transcriptome sequencing. In brief, mRNA was enriched using the NEBNext Poly(A) mRNA Magnetic Isolation Module (E7490, NEB, Ipswich, UK) from 3 µg of total RNA. Double-stranded cDNA was synthesized, and sequencing adaptors were ligated according to the manufacturer’s instructions (Illumina, San Diego, CA, USA). The ligated products were then purified with AMPureXP beads (Beckman Coulter, Brea, CA, USA) and were amplified for the construction of cDNA libraries. Library insert sizes ranged from 100 to 200 bp. The completed libraries were sequenced on the Illumina HiSeq 2000 platform. The original RNA-seq data was deposited into the Sequence Read Archive (SRA) of the National Centre of Biotechnology Information (NCBI) under the accession numbers SRR6015000, SRR6015001, and SRR5832159 to SRR58321162.

### 2.4. Data Processing and Assembly

Clean data (clean reads) were generated by removing reads containing adapters and poly-A as well as low quality reads from the raw data (raw reads) [20]. All of the downstream analyses were based on clean data of high quality. De novo assembly was carried out with the program Trinity using clean reads [21]. The high-quality transcripts were obtained after filtering and assembling. These transcripts were utilized for the further process of sequence clustering with Corset to create unigenes [22]. Next, a follow-up analysis was carried out with these unigenes, which were assembled from scratch. The published transcriptomes of other *Pinus* species from the NCBI non-redundant (Nr) [23], nucleotide sequences (Nt), protein family (Pfam), protein sequence database (Swissprot, [24], Gene Ontology (GO) [25], euKaryotic Ortholog Groups (KOG) [26], and the Kyoto Encyclopedia of Genes and Genomes (KEGG) databases [27] were used as references to obtain functional annotation information of unigenes by performing BLASTX with a cut-off *E* value of the best hit of ≤10^−5^.

### 2.5. Differential Expression Analysis (DEG)

Read counts of genes were calculated using RNA-Seq by Expectation-Maximization (RSEM) software [28]. The above results were translated into FPKM values (expected number of Fragments Per Kilobase of transcript sequence per Millions base pairs sequenced), which is currently the most commonly utilized method for estimating gene expression levels [29]. Differential gene expression between the two different libraries (female and male) was analyzed by DESeq [30]. The false discovery rate (FDR) was adjusted by *q*-values. The thresholds value of differentially expressed genes was set as *q* < 0.005 and log_2_ (fold change) > 1. The GO and KEGG functional enrichment analysis of DEGs was implemented to show the main biochemical and signal transduction pathways.

### 2.6. Identification of Expressed Sequence Tag-Simple Sequence Repeats (EST-SSRs)

We used the program MIcroSAtellite (MISA) [31] to detect potential EST-SSRs from unigenes in *P. bungeana*. The determining criteria were as follows: at least five repeats for the di-, and four repeats for the tri-, tetra-, penta- and hexanucleotide motifs. In addition, we used the software Primer 3 [32] to design the EST-SSR primer pairs. The setting parameters were the product size of PCR ranging from 100 to 300 bp, GC content of the primer from 40–60%, the length of primer ranging from 18–25 bp, and melting temperature from 50–65 °C. PCR amplification of SSR markers was performed in a 10 μL reaction volume, including 30–50 ng template DNA, 5 μL 2× Taq PCR Master Mix (Runde, Xi’an, China), 1 μL 2.5 μM of each primer, and 4 μL sterile water. The PCR procedure included an initial denaturation of 94 °C for 4 min, followed by 32 cycles of 45 s at 94 °C, 40 s at an annealing temperature of 50–60 °C for each primer, and 45 s at 72 °C, ending with a final extension of 5 min at 72 °C. We detected and validated the PCR products using silver-stained nondenaturing polyacrylamide gels [33]. In addition, we determined the allele sizes of each SSR genetic marker using the program Quantity One (Bio-Rad, Hercules, CA, USA).

### 2.7. Validation of EST-SSR Markers and Population Genetic Analysis

To assess the polymorphism of EST-SSR primers in *P. bungeana*, we randomly selected 84 pairs of primers to amplify genomic DNA. We used MICROCHECKER v2.2.3 to test the presence of null alleles for all loci [34]. The program ARLEQUIN v.3.11 [35] was implemented to detect loci violating assumptions of neutrality. The population genetic parameters, including the number of alleles (*N*_A_), observed heterozygosity (*H*_O_), and expected heterozygosity (*H*_E_) were calculated using the software POPGENE v.1.32 [36]. We also used the software GenAlEx 6 [37] to detect the Hardy-Weinberg equilibrium (HWE). The source of genetic variation was analyzed with AMOVA (Analysis of Molecular Variation) in ARLEQUIN v.3.11 [34]. The UPGMA (unweighted pair-group method with arithmetic averaging) analysis that was based on Nei’s (1987) [38] genetic distances among populations was performed using Mega software [39]. In addition, STRUCTURE was used to infer the population structure with an admixture model based on the Bayesian clustering approach [40]. The population genetic clusters (*K*) ranged from 1–10, and 10 independent runs were performed for each *K* with 10,000 burn-in and 100,000 Markov chain Monte Carlo (MCMC) replicates. The most likely number of *K* clusters was estimated using the Δ*K* statistics method with the program STRUCTURE HARVESTR [41].

## 3. Results

### 3.1. Transcriptome Sequencing and De Novo Assembly of Pinus bungeana

A total of 39.62 Gb high quality sequencing data (clean reads) were generated from six samples (female conelet 1, male conelet 1, female conelet 2, male conelet 2, female conelet 3, and male conelet 3) in *P. bungeana*. Among the sequencing datasets of female and male types, more than 96.14% and 96.59% of bases had a *q*-value > 20. The mean GC contents were 44.63% and 44.33% for female and male conelets, respectively, which suggests that the results of sequencing were relatively good (Table 2).

Using the Trinity software to assemble clean reads, we obtained a total 85,305 unigenes that were assembled with a mean length of 1199 bp and an N50 value of 1942 bp for *P. bungeana*, and the GC content was 44.48%. Most of the genes were relatively longer, with 34,343 (40.26%) unigenes greater than 500 bp. The size distribution of assembled unigenes was presented in Figure S2. The above results suggested that the quality of transcriptome sequencing and de novo assembly was relatively good, which were good enough to be used to carry out the subsequent bioinformatics analysis.

### 3.2. Gene Annotation of Pinus bungeana

**Figure 1:**
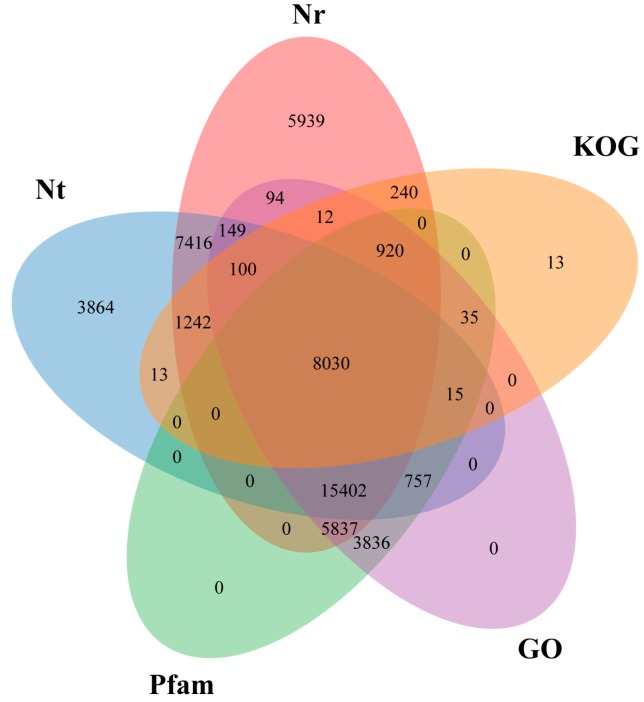
Venn diagram of annotation against the National Center for Biotechnology Information (NCBI) non-redundant (Nr), nucleotide sequences (Nt), Protein family (Pfam), Gene Ontology (GO) and euKaryotic Ortholog Groups (KOG) databases for the *Pinus bungeana* unigenes.

The assembled unigenes of *P. bungeana*, of which 53,944 (63.23%) were aligned and annotated with Nr, Nt, Pfam, Swissprot, KOG, GO, and KEGG databases, where 45,381 (53.19%), 36,988 (43.35%), and 34,319 (40.23%) unigenes had significant matches in Nr, Nt, and a manually annotated and Swissprot, respectively. Among all of them, 8030 unigenes could be common mapped by Nr, Nt, Pfam, GO, and KOG (Figure 1). The top-hit species in the annotated distribution was *Picea sitchensis* (18,903), followed by *Amborella trichopoda* (5203), and *Nelumbo nucifera* (3280) (Figure S3). The remaining 31,361 potential unigenes showed no homology to known sequences that are deposited in these databases.

**Figure 2:**
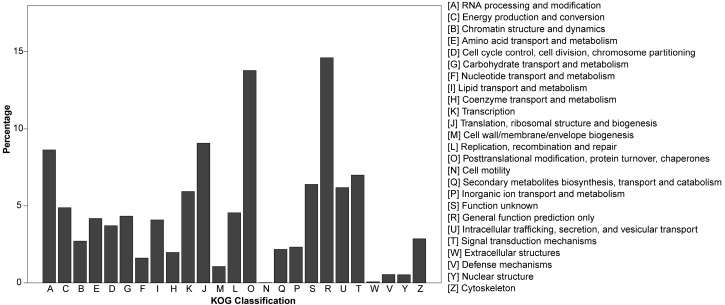
KOG functional classification of *P. bungeana* unigenes.

Based on the alignments, a total number of 10,620 (12.44%) annotated unigenes were identified from KOG. The database represents an attempt at phylogenetic classification of proteins encoded in complete genomes. Among the 25 KOG categories, the cluster in the assembly of male and female libraries for “General function prediction only” represented the largest group, followed by the “Posttranslational modification, protein turnover, chaperones”, “Translation, ribosomal structure and biogenesis” and “RNA processing and modification” clusters (Figure 2).

Furthermore, analysis of the GO categories showed that most of the unique sequences (35,187, 41.24%) were mapped to biological processes, followed by the molecular functions, and the least was the cellular component. The cellular process (19,470, 55.33%), binding (20,269, 57.60%), and cell (10,112, 28.74%) were the largest highly represented categories in these three main ontologies (Figure S4). All of the assembled unigenes were further annotated based on KEGG pathways. There were 15,721 (18.82%) unigenes that were divided and mapped into 19 functional pathways, with the ‘Metabolism’ cluster representing the largest group (Figure S5).

### 3.3. Analysis of Differently Expressed Genes in Male and Female Conelets of Pinus bungeana

We tested the global quality of the RNA-seq dataset by checking the reproducibility between each pair of samples (female and male conelets) [42], and found that reproducibility among the technical replicates of the gene expression was generally high (Figure S6).

Through the screening of differentially expressed genes (standard = 2X and FDR < 0.05), 12,073 unigenes were identified as differentially expressed between male and female conelets, which was comprised of 5766 unigenes that were up-regulated and 6307 unigenes that were down-regulated (Figure S7). Differential expression analysis indicated that there were more significant differences between the inter-gender than intra-gender variations by the three biological replicates from the female and male conelets, respectively (Figure S8). Moreover, we performed an enrichment analysis of GO and KEGG terms for these genes. For the GO term enrichment of DEGs, the six most highly represented terms were ‘metabolic process’, ‘catalytic activity’, ‘single-organism process’, ‘single-organism metabolic process’, ‘oxidation-reduction process’, and ‘oxidoreductase activity’ (Figure S9).

To further investigate the biological pathways that are active in sexual dimorphism, KEGG enrichment was performed based on the DEGs. The results indicated that 981 DEGs were significantly enriched in the top 20 KEGG pathways (*q*-value < 0.05), such as the 113 unigenes distributed in plant hormone signal transduction, the 132 unigenes allocated in starch and sucrose metabolism, and the 110 unigenes assigned to photosynthesis metabolism (Figure S10). DEGs in plant hormone signal transduction encoding the *Arabidopsis* response regulators (*ARR*), small auxin-up RNA (*SAUR*), and ethylene response factor (*ERF*) participated in the regulation of several hormone homeostasis and reproductive processes. These DEGs were involved in tryptophan metabolism (Ko00380), zeatin biosynthesis (Ko00908), cysteine, methionine (Ko00270), gibberellin (GA) pathway (Ko00904), carotenoid biosynthesis (Ko00906) and brassinolide (BR) pathway (Ko00905) (Figure S11, Table S2). Furthermore, we found that the female-biased cinnamoyl-CoA reductase (*CRR*) (EC: 1.2.1.44) was enriched in the phenylpropanoid biosynthesis pathways (Ko00940) (Figure S12, Table S2). The general phenylpropanoid components have been well shown to affect pollen development and male sterility [43]. Overall, these annotations provided a substantial resource for investigating specific processes, functions, and pathways during sexual determination.

### 3.4. Polymorphism of EST-SSR Markers and Population Genetic Structure

**Figure 3:**
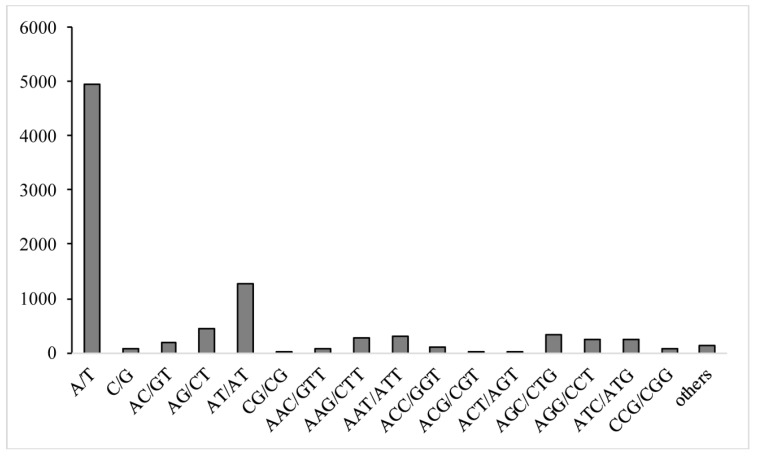
The numbers and motifs of simple sequence repeats (SSR) of *P. bungeana* unigenes.

With the purpose of developing novel molecular markers, the 85,305 unigenes of *P. bungeana* were used to mine for potential SSR markers. In total, 8791 SSRs were detected in 7859 unigenes. The most abundant repeat types were mononucleotides (5019), followed by dinucleotides (1926) and trinucleotides (1711). The dominant classes of the sequence repeat were A/T (4942) in the mononucleotides and AT/AT (1283) in the dinucleotides (Figure 3).

To verify the reliability of these SSR primers, we randomly selected 84 pairs of primers to amplify the genomic DNA of *P. bungeana*. Sixty-four individuals of six natural populations of *P. bungeana* were sampled to determine the polymorphism of these microsatellite markers. Among these, 84 primer pairs, 40 resulted in successful PCR amplification and showed the predicted PCR products, 19 of which showed polymorphisms among 64 individuals (Table 1 and Table S1, Figure S1). We found no evidence for the existence of null alleles. Two EST-SSR loci (1314 and 22,642) departed significantly from the simulated *F*_ST_ distribution, indicating that they could be under disruptive selection or linked to a locus under selection. These two loci were therefore removed in subsequent analysis. The mean number of alleles (*N*_A_), observed heterozygosity (*H*_O_), and expected heterozygosity (*H*_E_) ranged from 1.333–3, 0.033–0.492, and 0.067–0.455 for each loci, respectively (Table 3). For each population, the mean value of the number of alleles (*N*_A_), observed heterozygosity (*H*_O_), and expected heterozygosity (*H*_E_) ranged from 1.579–1.842, 0.112–0.256, and 0.168–0.240, respectively (Table 3 and Table 4). The probabilities of deviation from the HWE proved that most of the EST-SSR markers significantly violated the HWE (Table 3). Population genetic differentiation was also significant across all loci (*F*_ST_ = 0.252 ***) (Table S3). An Unweighted Pair Group Method with Arithmetic Mean (UPGMA) dendrogram that was based on Nei’s genetic distance showed that all of the populations were divided into two different clusters, cluster I for populations W, A, Q, and Y, and cluster II, for populations C and L (Figures S13 and S14).

**Figure 4:**
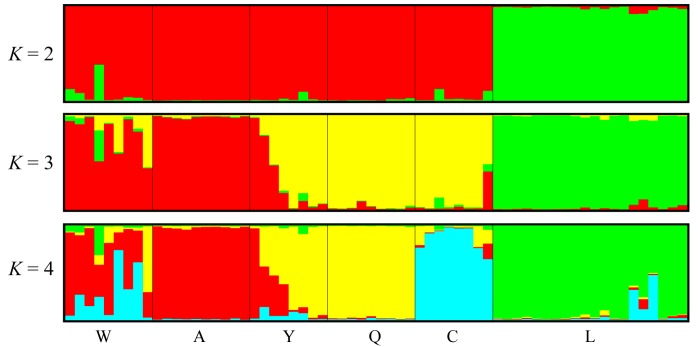
Bayesian clustering analysis of population structure of *P. bungeana*.

For the population genetic structure of *P. bungeana*, the most likely population cluster K was two with the STRUCTURE analysis (Figure S13). The six populations of *P. bungeana* were assigned into two distinct groups: one group included the populations W, A, Y, Q, and C, while the other included only population L (Figure 4). At *K* = 4, samples from population C were further subdivided into an independent group. This is consistent with the UPGMA dendrogram.

## 4. Discussion

### 4.1. Transcriptome Characterization

Transcriptome sequencing is an effective and rapid method to identify genomic resources for non-model plants [7], especially for the conifer species, which possess complex and large genomes [6,13]. In this study, a total of 39.62 Gb clean reads were obtained from the transcriptome sequencing of the conifer species *P. bungeana*. The total number of assembled unigenes (85,305) was more than that of the other conifer species *P. tabuliformis* (46,584 unigenes) [11], also the N50 length of the unigenes, 1942 bp, was longer than that of *P. tabuliformis* (N50 = 744 bp) [11], which suggested that the assembly procedure for *P. bungeana* had good quality in this study. Intriguingly, few unigenes (1124, 2.48%) were annotated for an important *Pinus* species, *P. taeda*. This difference indicated the potential to discover novel genes that are specific to *P. bungeana*. Additionally, *Pinus taeda* belongs to Subgen. *Pinus*, whereas *P. bungeana* belongs to the Subgen. *Strobus* (Sweet) Rehd. The two *Pinus* species were divided into different groups, as well as different wild geographic distributions of two conifer species, which might lead to interspecific differences between them.

### 4.2. The Pathway Analysis of Differentially Expressed Genes

#### 4.2.1. The Plant Hormones Pathway Analysis

Plant hormones are endogenous regulators with multiple signal functions that affect nearly all aspects of plant growth and development [44,45,46]. Some studies have indicated that various phytohormones were likely to participate in the regulation of sex-determining genes and developmental pathways in unisexual flowers [47]. For example, some researchers have found that plant hormones and gibberellins (GAs) could promote early flowering in conifers and enhance the regularization of seed production [48,49,50]. In addition, the auxin-responsive protein (IAA), ethylene, and kinetin could also elicit a feminization effect on the sex of hemp [51]. In our study, the signaling pathways of several hormones, including auxin (Tryptophan metabolism, Ko00380), ethylene (cysteine and methionine metabolism, Ko00270), and cytokinin (zeatin biosynthesis, Ko00908), steroid hormones (brassinolide pathway, Ko00905) were enriched by pathway-based analysis (Figure S10). The characterization and future analysis of critical genes responsible for plant hormone production and signaling would greatly facilitate studies on the complex genetic network of sexual differentiation in *P. bungeana*.

#### 4.2.2. Tryptophan Metabolism

Tryptophan (TRP)- dependent metabolism (Ko00380) is considered to be one of the main pathways of auxin (*IAA*) biosynthesis [52]. The primary auxin response genes consisted of members of three gene families, the auxin influx carrier (*AUX/IAA*), small auxin up RNA (*SAUR*), and gretchen hagen3 (*GH3*) [53], which may participate in sex differentiation in *P. bungeana* (Figure S11, Table S2). The auxin-signaling pathway, as mediated by *AUX1* and auxin response factor (*ARF*), was up-regulated in female conelets of *P. bungeana*. The auxin influx carrier (Cluster-2735.16335 (3.2832)) (belonging to the AUX1 LAX family) has been demonstrated to encode a high-affinity auxin influx carrier and plays a major role in many aspects of plant growth and development [54]. ARF (Cluster-2735.24659 (2.3325)), such as ARF6 and ARF8 were required to promote inflorescence stem elongation and late stages of petal, stamen, and gynoecium development in *Arabidopsis thaliana* [53]. In addition, it also has a conserved role in controlling the growth and development of vegetative and flower organs [55]. Therefore, the function of this auxin influx carrier (Cluster-2735.16335 (3.2832)) and auxin response factor (Cluster-2735.24659 (2.3325)) in sex differentiation is worthy of further study and exploration.

#### 4.2.3. Cysteine and Methionine Metabolism

Ethylene has an extensive regulation role in the plant growth and development process, especially in the aspect of sexual differentiation [56,57]. In this pathway (Ko00270), one unigene assembly (Cluster-2735.8266 (-1.8084)) was annotated to serine/threonine-protein kinase (CTR1), which showed an even closer similarity to the *A.s thaliana CTR1* gene (Figure S11, Table S2). The ethylene receptor (*ETR2*) acts upstream of *CTR1*, coding for a Raf-related protein kinase, which is ubiquitously expressed and has a higher level of expression in some tissues, including inflorescence, floral meristems, petals, and ovules [58].

#### 4.2.4. Zeatin Biosynthesis

Cytokinins (CTK) are another key plant hormone to affect plant gender expression, which comes from zeatin biosynthesis (Ko00908) (Figure S11). The cytokinin signal is perceived by three membrane-located receptors named *Arabidopsis* histidine kinase 2 (AHK2), AHK3, and AHK4/CRE1 [59]. These receptors in the sporophyte are indispensable for anther dehiscence, pollen maturation, the induction of pollen germination by the stigma, and female gametophyte formation and maturation [60]. In our study, the histidine kinase 2-like (Cluster-2735.33324 (3.6499)), histidine kinase 3 (Cluster-2735.50860 (5.7972)), histidine kinase 4 isoform X2 (Cluster-2735.21997 (3.1149)), and the histidine kinase 4 isoform X1 (Cluster-2735.22398 (6.2557)) were identified and up-regulated in females. Furthermore, the histidine-containing phosphotransfer protein (AHP) (Cluster-2735.25394 (7.0138)) was also up-regulated in females, which was the mediator in a multistep phosphorelay pathway for cytokinin signaling [61]. It also negatively regulates the thickening of the secondary cell wall of the anther endothecium [61]. These results suggested that CTK was expected to play important roles of inducing female in the sexual differentiation of *P. bungeana*. A total of 15 unigenes encoding ARRs were enriched (Table S2). There was a functional overlap among the ARRs, which can act as positive regulators of cytokinin signal transduction [62]. These genes might be useful in identifying the system that induced sexual differentiation and possibly respond to the levels of cytokinin established *P. bungeana*.

#### 4.2.5. Other Important Plant Hormones

Carotenoid is a precursor of abscisic acid (ABA), which plays an extremely important role, as well as gibberellin, on regulating plant growth and development [63]. Research has shown that ABA has certain effects on affecting the plant sex expression [64,65]. As two types of receptors of ABA, PYRABACTIN RESISTANCE1 (PYR1)/PYR1-LIKE (PYL) (Cluster-2735.52019 (–11.2) and Cluster-2735.52803 (–8.8097)) were up-regulated in males. In addition, Type 2C protein phosphatases (PP2Cs) (Cluster-2735.44601 (–7.1102) and Cluster-2735.38390 (–1.6378)) were also increased in males, which are vitally involved in ABA signaling (Figure S9, Table S2) [66]. ABA binds to PYR1, which, in turn, binds to and inhibits PP2Cs [66]. In summary, ABA perception by PYR/PYLs plays a major role in the regulation of seed germination and establishment, the basal ABA signaling that is required for vegetative and reproductive growth, stomatal aperture, and transcriptional response to the hormone [67].

Gibberellin (GA) derived from the diterpenoid biosynthesis pathway (Figure S11) is expected to influence plant sexual development. For example, in the monoecious species of *Buchloe dactyloides*, GA showed a dual effect with the induction of males and inhibition of females [68]. GA could increase the male-induced trait as the concentrations increased in the seedling of *Spinacia oleracea* [69]. Gibberellin is perceived by its nuclear receptors GA INSENSITIVE DWARF1s (GID1s), which then trigger the degradation of downstream repressors DELLAs [70]. In our study, the gibberellin receptor GID1 (Cluster-2735.41544 (4.7043)) was up-regulated in female conelets. The functional study of GID1 mutant combinations confirmed that GID1A plays a major role during fruit-set and growth, whereas GID1B and GID1C have specific roles in seed development and pod elongation, respectively [69]. GID1A was expressed throughout the whole pistil, while GID1B was expressed in ovules, and GID1C was expressed in valves. In our study, we observed that the gibberellin receptor GID1C (Cluster-2735.47107 (–3.2955) were up-regulated in male conelets (Table S2). GA perception by GID1 causes slender rice1 (SLR1) protein degradation involving the F-box protein GID2; this triggers GA-associated responses, such as shoot elongation and seed germination [71]. There were four genes that were annotated as F-box proteins GID2 (Cluster-2735.64327 (1.9978), Cluster-2735.21997 (3.1149), Cluster-2735.60058 (Inf), and Cluster-4222.0 (Inf)) were up-regulated in female conelets.

The brassinosteroids (BR) are the most important discovery after GA as plant growth regulators [72]. BR signaling establishes an unexpected genetic pathway in the floral-regulating network [72]. Two genes encoding protein brassinosteroid insensitive 1 (BRI1) (Cluster-2735.63413 (2.7201) and Cluster-2735.44795 (1.7374)) of *P. bungeana* were involved in the brassinolide synthesis pathway and both showed high levels of expression in female conelets (Figure S11, Table S2). BRI1 was found to have the predominant function as a flowering-time enhancer, and also exhibited the elevated expression of potent floral repressor FLOWERING LOCUS C (FLC) [73].

Jasmonate (JA) was synthesized by the free α-linolenic acid through the lipoxygenase (LOX) pathway [74], which is necessary for the normal development of floral organs. The biosynthesis and signal transduction process of jasmonate directly influences the developmental status of the flower organ. These studies have shown that jasmonate is mainly involved in the regulation of plant development of stamens in male flower organ [75,76,77,78,79,80]. Transcription factor MYC2 (Transcription factor MYC2-like, Cluster-2735.41360 (2.4305)) that is involved in the pathway of jasmonate synthesis was identified [80], which was up-regulated in female conelets in *P. bugeana* (Table S2). MYC2 is involved in JA-regulated plant development, lateral and adventitious root formation, flowering time, and shade avoidance syndrome [81]. Another of its notable functions is to regulate the crosstalk between the signaling pathways of JA and those of other phytohormones, such as ABA, GAs, and IAA [81].

This study preliminarily explored the co-expression of various hormones through multiple metabolic pathways and mechanisms in *P. bungeana*. In conclusion, each type of plant hormone did not independently regulate plant sexual differentiation, but a variety of hormones formed a network control mode to influence each other (Figure S11). Nearly all kinds of plant hormones could affect gender differentiation to a certain extent. However, it is important to note that the hormone role of plant sex expression is also not absolute. For example, plant hormones are hardly involved in *Silene* (*Silene latifolia*) flower development [82]. In addition, the regulation of plant hormones in female or male organs is related to the plant species, but the same hormones could have completely opposite effects in different plants [83,84].

#### 4.2.6. Photosynthesis Metabolism Pathway

We also found numerous genes in the phenylpropanoid biosynthesis pathway (Ko00940) that were differentially expressed between the two sexes (Figure S12, Table S2). Significant male expression occurred in genes encoding caffeoyl shikimate esterase (CSE, EC: 3.1.1), and ferulate-5-hydroxylase (F5H, EC: 1.2.1.44). Several genes in the phenylpropanoid pathway also exhibited significantly higher transcript abundance in females, including shikimate *O*-hydroxycinnamoyltransferase (HCT, EC: 2.3.1.133) and cinnamoyl-CoA reductase (CCR, EC: 1.2.1.44) (Figure S10, Table S2). Two genes that were annotated as F5H (Cluster-2735.14069 (-3.2707) and Cluster-2735.56444 (-Inf)) were significantly up-regulated in male conelets. Over-expressing F5H in a line of *Arabidopsis* lacking caffeic acid *O*-methyltransferase induced a new type of lignin enriched 5-hydroxy-guaiacyl units, which had a profound impact on plant growth and development and cell-wall properties, and resulted in male sterility due to the complete disruption of the formation of the pollen wall [85]. The differential expression of numerous genes (cinnamoyl-CoA reductase, CCR) and enzymes (shikimate *O*-hydroxycinnamoyltransferase, HCT; caffeoyl shikimate esterase, CSE) that are involved in lignin biosynthesis and metabolism in the phenylpropanoid pathways could be used to further reveal the link between the biosynthetic pathways of lignin and the pollen wall-forming sporopollenin [43,86].

### 4.3. Polymorphism of EST-SSR Markers and Population Structure of Pinus bungeana

In this study, 84 primer pairs of *P. bungeana* were randomly selected from 8791 primer pairs that were successfully designed for EST-SSR loci. Within these primer pairs, 40 (47.6%) resulted in successful PCR amplification and showed clear bands with the predicted PCR products, and 19 (22.6%) primers showed polymorphism among the sampled 64 individuals. As the *Pinus* species are generally diploid plants [8], the failed amplification of EST-SSR markers might be primarily due to the highly repetitive sequences and unsuitable primer sequences in conifer species (e.g., Reference [6]). Furthermore, the average number of alleles (*N*_A_) of 1.723 across the six populations of *P. bungeana* was lower than that reported for other gymnosperms (Table 4). These results suggested that the polymorphism level of the 19 EST-SSR markers that was developed in this study was low to moderate when compared with other conifer species [87,88].

Generally, the patterns of genetic variation and population structure are the basis of species evolution [89,90]. The evolutionary potential and adaptive ability of a natural species are largely dependent on the levels and distributions of its genetic diversity [91,92]. In this study, we detected a low level of genetic variability (an average value of *H*_O_ = 0.206, *H*_E_ = 0.205) at the species level for the endemic conifer. The reason for the low diversity may be due to the limited sample size of *P. bungeana* in this study. In addition, the dramatic changes of population size, genetic drift, and long generation time of species might have caused the low genetic diversity [93]. In a previous study, Yang et al. [17] concluded that the natural populations of *P. bungeana* experienced dramatic range fluctuations based on three nuclear genes. In the evolutionary history of *Pinus*, genetic drift and population size changes that were caused by climatic oscillations and geological events may have caused the low genetic variability. Meanwhile, the long generation time of the *Pinus* species and evolution process with low mutation rate may have led to the low level of diversity of *P. bungeana* [90]. On the other hand, in recent years, habitat fragmentation and human over-cutting may also have affected the genetic variation of natural populations of *P. bungeana* [17].

In addition, the management and protection of wild natural populations are very important to the endemic conifer species. Priority should be placed on conserving populations with high levels of genetic diversity; for example, population Y of *P. bungeana* (*H*_O_ = 0.255, *H*_E_ = 0.240, with an average value of *H*_O_ = 0.206, *H*_E_ = 0.205), which had the highest diversity should be of the highest priority (Table 4, Figure 4). In addition, the cluster analysis revealed two distinct genetic groups for the sampled six populations in this endemic species, which may have been caused by the fragmentation of habitats and long term geographic isolation among the different populations. According to the STRUCTURE results, samples from population C were further subdivided into an independent group at K = 4, which was consistent with the UPGMA dendrogram. It seems that although a distinct genetic structure was identified in *P. bungeana*, we also need to further estimate genetic diversity and population structure to obtain accurate conclusions based on more samples from different natural populations.

## Figures and Tables

**Table 1:** Information on the geographical distribution of wild populations of *Pinus bungeana*.

Code	Location	Longitude (E)	Latitude (N)	Altitude (m)	*N*
W	Wuzi Mountain, Shaanxi	107.83	32.93	700	9
A	Ankang, Shaanxi	108.94	32.97	577	10
Y	Huozhou, Shanxi	111.87	36.60	900	8
Q	Qinyang, Henan	112.79	35.22	972	9
C	Taiyuan, Shanxi	112.32	37.72	1100	8
L	Mianyang, Sichuan	105.09	32.22	778	20

Note: *N* = number of individuals.

**Table 2:** Results of quality statistics of transcriptome database of *Pinus bungeana*.

Sample	Raw Reads	Clean Reads	Clean Bases (bp)	Q20 (%)	Q30 (%)	GC (%)
Female 1	56000000	54951878	6.87G	96.14	92.25	44.26
Male 1	53506322	52712306	6.59G	96.64	93.36	44.05
Female 2	56000000	55262192	6.91G	96.74	93.48	44.28
Male 2	54024824	53089856	6.64G	96.63	93.28	44.32
Female 3	41215900	39528272	5.93G	96.69	91.84	45.35
Male3	46220380	44510796	6.68G	96.59	91.64	44.63

Note: Q20 = the percentage of bases with a phred value >20; Q30 = the percentage of bases with a phred value >30.

**Table 3:** The parameters of genetic diversity of the 17 expressed sequence tag-simple sequence repeats (EST-SSR) primers of *P. bungeana*.

Primers	*N*	*N*_A_	*N*_E_	*H*_E_	*H*_O_	*P*
5358	64	1.5	1.216	0.134	0.188	0.518
7309	64	1.833	1.528	0.294	0.208	0.021 *
24177	64	1.667	1.236	0.173	0.164	0.235
67970	64	1.333	1.148	0.097	0.126	0.634
10335	64	1.5	1.108	0.087	0.033	0.000 ***
73317	64	2	1.568	0.350	0.489	0.009 **
72763	64	1.5	1.32	0.189	0.208	0.033 *
66538	64	2	1.169	0.112	0.045	0.000 ***
60339	64	1.5	1.078	0.067	0.072	0.835
34533	64	1.667	1.494	0.262	0.413	0.049 *
33255	64	1.333	1.314	0.161	0.158	0.000 ***
10373	64	1.5	1.205	0.130	0.135	0.025 *
11371	64	1.667	1.191	0.117	0.056	0.000 ***
19808	64	1.833	1.583	0.339	0.492	0.008 **
7028	64	1.667	1.096	0.083	0.089	0.000 ***
6545	64	3	2.404	0.455	0.345	0.072
7029	64	1.833	1.559	0.321	0.240	0.988
Mean		1.754	1.383	0.205	0.206	

Note: *N* = number of individuals; *N*_A_ = number of alleles; *N*_E_ = number of effective alleles; *H*_E_ = expected heterozygosity; *H*_O_ = observed heterozygosity; *P* = Tests for Hardy-Weinberg Equilibrium (* *P* < 0.05, ** *P* < 0.01, *** *P* < 0.001).

**Table 4:** Genetic diversity parameters of six natural populations of *P. bungeana*.

Population	*N*	*N*_A_	*N*_E_	*H*_E_	*H*_O_
W	9	1.789	1.450	0.231	0.231
A	10	1.632	1.310	0.189	0.256
Y	8	1.842	1.510	0.240	0.255
Q	9	1.842	1.436	0.221	0.187
C	8	1.579	1.292	0.168	0.197
L	20	1.842	1.298	0.181	0.112
Mean		1.754	1.383	0.205	0.206

## Supplementary Materials

The following are available online at www.mdpi.com/2073-4425/8/12/393/s1. Figure S1: The geographical distributions of natural populations of *Pinus bungeana*. Figure S2: Length distributions of *Pinus bungeana* transcripts and unigenes. Figure S3: The number and distribution of homologous unigenes in *Pinus bungeana*. Figure S4: Gene Ontology (GO) classification of *Pinus bungeana* unigenes. Figure S5: KEGG functional classification of *Pinus bungeana* unigenes. Figure S6: Spearman correlation matrix of experimental replications of transcriptome sequencing datasets of *Pinus bungeana*. Figure S7: Analysis of differential unigenes expression. In total, 12,073 unigenes were identified as differentially expressed between male and female conelets, which comprised 5766 unigenes that were up-regulated and 6307 unigenes that were down-regulated. Figure S8: Venn diagram of differential expression unigenes within three biological replications from male and female conelets, respectively. Figure S9: Analysis of differential unigenes expression. Figure S10: KEGG pathways enriched for differentially expressed genes of male and female conelets in *Pinus bungeana.* Figure S11: Plant hormone signal transduction in *Pinus bungeana* was adapted from the Kyoto Encyclopedia of Genes and Genomes (KEGG) pathway. Figure S12: Cysteine and Methionine metabolism pathway was adapted from the KEGG. Figure S13: Results of the Bayesian assignment analysis of *Pinus bungeana* using the program STRUCTURE. Figure S14: Dendrogram for natural six populations of *Pinus bungeana* based on 17 SSR loci. Table S1: Information of SSR Primers of *Pinus bungeana* used in this study. Table S2: Differently expressed genes in plant hormone signal transduction and photosynthesis metabolism pathways. Table S3: Results of the analysis of molecular variance (AMOVA) performed on six populations in *Pinus bungeana* using 17 microsatellite markers.
