# Supplementary material for: Comparative Transcriptome Analysis of Male and Female Conelets and Development of Microsatellite Markers in Pinus bungeana, an Endemic Conifer in China
Source: Genes (Basel). 2017 Dec 19;8(12):393. doi: 10.3390/genes8120393 (PMC5748711; doi:10.3390/genes8120393)
Supplement: Supplementary file 1 [file genes-08-00393-s001.pdf]

Comparative Transcriptome Analysis of Male and Female Conelets and Development of Microsatellite Markers in *Pinus Bungeana*, an Endemic Conifer in China

Dong Duan [#], Yun Jia [#], Jie Yang, and Zhong-Hu Li*

Key Laboratory of Resource Biology and Biotechnology in Western China, Ministry of Education, College of Life Sciences, Northwest University, Xi'an 710069, China

[#] These two authors contributed equally to this work

* Correspondence: lizhonghu@nwu.edu.cn; Tel: +86 29 88302411; Fax: +86 29 88302411

Figure legends

Figure S1. The geographical distributions of natural populations of *Pinus bungeana.*

Figure S2. Length distributions of *Pinus bungeana* transcripts and unigenes.

Figure S3. The number and distribution of homologous unigenes in *Pinus bungeana*.

Figure S4. Gene Ontology (GO) classification of *Pinus bungeana* unigenes.

Figure S5. KEGG functional classification of *Pinus bungeana* unigenes. The results are summarized in five main categories: A, Cellular Processes; B, Environmental Information Processing; C, Genetic Information Processing; D, Metabolism; E, Organismal Systerms.

Figure S6. Spearman correlation matrix of experimental replications of transcriptome sequencing datasets of *Pinus bungeana*. The numbers represent the correlation coefficients between the samples. Heat maps represents samples similarity of expression patterns with correlation coefficients.

Figure S7. Analysis of differential unigenes expression. In total, 12,073 unigenes were identified as differentially expressed between male and female conelets, which comprised 5,766 unigenes that were up-regulated and 6,307 unigenes that were down-regulated.

Figure S8. Venn diagram of differential expression unigenes within three biological replications from male and female conelets, respectively.

Figure S9. Analysis of differential unigenes expression. BP, MF and CC represent biological processes, molecular functions, and cellular component, respectively. In total, 12,073 unigenes were identified as differentially expressed between male and female conelets, which comprised 5,766 unigenes that were up-regulated and 6,307 unigenes that were down-regulated.

Figure S10. KEGG pathways enriched for differentially expressed genes of male and female conelets in *Pinus bungeana.*

Figure S11. Plant hormone signal transduction in *Pinus bungeana* was adapted from the Kyoto Encyclopedia of Genes and Genomes (KEGG) pathway available online (http://www.genome.jp/kegg/pathway.html). A total of 113 differentially expressed genes in plant hormone signal transduction, encoding arabidopsis response regulators (*ARR*), small auxin-up RNA (S*AUR*), and ethylene response factor (*ERF*), participated in the regulation of several hormone homeostasis and reproductive processes. These DEGs were involved in

tryptophan metabolism (Ko00380), zeatin biosynthesis (Ko00908), cysteine and methionine (Ko00270). Red represents up-regulated genes in female *vs* males, green represents downregulation genes in female *vs* males, and yellow represents up-regulated and down regulated genes in female *vs* males.

Figure S12. Cysteine and Methionine metabolism pathway was adapted from the Kyoto Encyclopedia of Genes and Genomes (KEGG) pathway available online (http://www.genome.jp/kegg/pathway.html). Many genes identified by KEGG as encoding cinnamoyl-CoA reductase 1 and 2 (*CRR1*/*CRR2*) (EC: 1.2.1.44) are enriched in *Pinus bungeana.* Red represents up-regulated genes in female *vs* males, green represents downregulation genes in female *vs* males, and yellow represents up-regulated and down-regulated genes in female *vs* males.

Figure S13. Results of the Bayesian assignment analysis of *Pinus bungeana* using the program STRUCTURE. The ΔK was plotted against various values of K, suggesting K= 2 as the most likely number of clusters.

Figure S14. Dendrogram for natural six populations of *Pinus bungeana* based on 17 SSR loci.

Table S1. Information of SSR Primers of *Pinus bungeana* used in this study.

Primers	Repeats unit	Sequence (5'-3')	Tm
1314	(GAGGAA)4	GAGGAGGCAAATGAGCAAAG	60.0
		TTAGTCGCTTCTTTTGCGGT	
5358	(CAACCT)4	GCATTCGAATTCCTCTCAGC	60.0
		TCGGATACTTCGGGTTCTTG	
7309	(CTG)6	TTGCTGCTGCTGTAACTGCT	60.0
		TGCAGCAGGTTCCCTCTAGT	
24177	(GGCTGC)4	CTGGGGAGTATGCACACCTT	60.0
		CAGTATCAACAGCAAGCCCA	
67970	(GAC)8	AGCGAACGGATGAAAGATTG	60.0
		GTGAGGAAGCCAAGTTGGAG	
10335	(TTGAT)4	CAACGTGGCCTCTGAGAAAT	60.0
		AGGGTTGCCTTCTTTCCAAT	
73317	(TAATCC)4	AACGACATCGACAACGACAA	58.0
		CCACGTGGTTTGTTGTGAAC	
72763	(AAAACC)4	GGCAATTCTGCAGTAGCCTC	58.0
		ATGGTCTGTCCATTTCGGTG	
66538	(GGGCGA)4	ATATTGATCAGGCGAGGCAG	58.0
		GGATTGTTGCAGGTTTTCGT	
60339	(ACATAT)4	TCTCAATTAAGCGGACAAGTTACA	58.0
		AGCCCTCTGTGATTGGACTC	
34533	(CTCACC)6	ATCTCGGCCAATTTGTCATC	58.0
		TTGGTCCACCTTTCATCCTC	
22642	(ATT)5(ATC)6	CCGTGTGCTTGATTGATCTG	58.0
		TCCTGTTTGTAATGGTTGCG	
33255	(AAGGC)5(GAG)5	TCAGCAACCAAACCATACCA	58.0
		TGCACTCGCTCCCTATCTTT	
10373	(GCAGGG)5	GGCTTGCATCCATCAATTCT	58.5
		GAGGCTGGAGCTCTGTGAAC	
11371	(AAATA)5	CAATTCGCGGTGTGATTATG	58.0
		TTCATCGAGCATGGAAACAG	
19808	(CTGTG)5	CTCATTCTTCCGCCATTGTT	58.0
		AGAGGCACAGAACAGCACAA	
7028	(TTC)8	AGCCATTTCTTCTGCTTCCA	58.0
		TTTTCACCCATTCTCCTTCG	
3534	(AT)12	AAGCATCTGCACCTATTGGG	58.0
		GTGGAATTGAGATCGGCTGT	
10962	(TA)11	CGGCCTTTCACTTCTGGTAG	58.0
		TGCTGACAAACAAACCGAGA	

Note: Tm = optimal annealing temperature

Table S2. Differently expressed genes in plant hormone signal transduction and photosynthesis metabolism pathways.

KEGG		Genes	Up-regulate genes (Female)	Down-regulated (female)
plant hormone signal				
transduction	Tryptophan metabolism	SAUR	Cluster-8494.0(Inf)	Cluster-2735.2887(-5.1571)
			Cluster-2735.18221(Inf)	Cluster-2735.14081(-5.5712)
			Cluster-2735.51357(4.6422)	Cluster-2735.56717(-8.4844)
			Cluster-2735.60733(Inf)	Cluster-2735.2956(-10.139)
			Cluster-2735.7773(3.8687)	Cluster-2735.56511(-7.4818)
			Cluster-2735.7774(Inf)	Cluster-2735.10750(-5.9766)
			Cluster-3801.0(Inf)	Cluster-2735.54045(-9.4224)
			Cluster-2735.57879(2.3789)	Cluster-2735.52807(-9.0952)
			Cluster-2735.22648(Inf)	Cluster-2735.52806(-Inf)
			Cluster-707.1(Inf)	Cluster-2735.61932(-Inf)
			Cluster-2735.56813(Inf)	Cluster-2735.7778(-6.803)
				Cluster-2735.7777(-Inf)
				Cluster-2735.12488(-7.926)
				Cluster-2735.2688(-2.584)
				Cluster-2735.52641(-11.003)
				Cluster-2735.53633(-Inf)
				Cluster-2735.12513(-2.6559)
				Cluster-2735.65134(-Inf)
				Cluster-2735.12180(-8.4152)
			Cluster-2735.59965(5.3644)	
		AUX1	Cluster-2735.16335(3.2832)	
		AUX/IAA	Cluster-2735.45441(4.2689)	Cluster-2735.18191(-2.2323)
			Cluster-2735.31633(3.2441)	Cluster-2735.18844(-5.7736)
			Cluster-2735.33273(3.8196)	
			Cluster-2735.28099(3.2635)	
			Cluster-2735.41715(2.8053)	
			Cluster-2735.53284(2.4338)	
			Cluster-2735.26404(4.1823)	
			Cluster-2735.26492(Inf)	
			Cluster-2735.26008(5.2944)	
			Cluster-2735.39031(3.1877)	
			Cluster-2735.32010(5.0073)	
		ARF	Cluster-2735.24659(2.3325)	
			Cluster-2735.22381(3.5426)	
			Cluster-2735.26026(3.2961)	
		GH3	Cluster-2735.13794(2.088)	Cluster-2735.62028(-7.7805)
	Zeatin biosynthesis	ARR-A	Cluster-2735.7083(5.4752)	Cluster-2735.57119(-Inf)
			Cluster-2735.44687(8.6534)	Cluster-2735.55866(-Inf)
				Cluster-2735.55867(-Inf)
				Cluster-2735.55864(-6.021)

			Cluster-2735.55865(-Inf)
			Cluster-2735.55861(-Inf)
			Cluster-2735.4392(-2.9454)
			Cluster-2735.9026(-7.7336)
	ARR-B	Cluster-2735.42790(2.6023)	Cluster-2735.19108(-4.2748)
		Cluster-2735.42791(2.621)	Cluster-2735.19107(-4.1435)
			Cluster-2735.19106(-3.2426)
	CRE1	Cluster-2735.50860(5.7972)	
		Cluster-2735.22397(5.0003)	
		Cluster-2735.22398(6.2557)	
		Cluster-2735.33324(3.6499)	
	AHP	Cluster-2735.25394(7.0138)	Cluster-2735.313(-3.2911)
Diterpenoid biosynthersis	GID1	Cluster-2735.41544(4.7043)	Cluster-2735.53965(-4.278)
			Cluster-2735.36203(-1.9156)
			Cluster-2735.47107(-3.2955)
	GID2		Cluster-2735.64327(1.9978)
			Cluster-2735.21997(3.1149)
			Cluster-2735.60058(Inf)
			Cluster-4222.0(Inf)
Carotenoid biosynthesis	PYR/PYL	Cluster-2735.31046(1.5835)	Cluster-2735.52019(-11.2)
		Cluster-2735.30469(1.665)	Cluster-2735.52803(-8.8097)
			Cluster-2735.4089(-3.4894)
			Cluster-2735.2978(-5.3043)
			Cluster-2735.12137(-Inf)
	PP2C	Cluster-2735.41544(4.7043)	Cluster-2735.53965(-4.278)
			Cluster-2735.36203(-1.9156)
			Cluster-2735.47107(-3.2955)
	SnRK2		Cluster-2735.10678(-Inf)
			Cluster-2735.27776(-2.6752)
			Cluster-2735.46194(-3.6695)
			Cluster-2735.46192(-3.5798)
	ABF		Cluster-2735.40065(-2.2889)
			Cluster-2735.40066(-1.5581)
Cysteine and methionine biosynthesis	ERF		Cluster-12842.0(-4.7991)
	CTR1		Cluster-2735.8266(-1.8084)
	MPK6		Cluster-2735.39420(-1.7773)
	EIN3	Cluster-2735.31382(1.7214)	
a-linolenic acid metobolism	MYC	Cluster-2735.41360(2.4305)	
	COI1	Cluster-2735.33185(1.9088)	Cluster-2735.27649(-2.4554)
		Cluster-2735.16735(1.7131)	
Brassinosteroid biosynthesis	BRI1	Cluster-2735.63413(2.7201)	
		Cluster-2735.44795(1.7374)	
	BSK		Cluster-2735.8266(-1.8084)

		BZR1/2	Cluster-2735.5826(4.1959)	
		YCYD3	Cluster-2735.35481(7.4523)	
			Cluster-2735.47801(9.5398)	
			Cluster-2735.42546(Inf)	
	Phenylalanine metabolism	NPR1	Cluster-8901.0(5.4807)	
		TGA	Cluster-2735.27941(2.3996)	
		PR-1		Cluster-2735.53111(-Inf)
Phenylpropanoid biosynthesis	phenylalanine ammonia-lyase	EC:4.3.1.24	Cluster-2735.35105(3.0094)	
			Cluster-2735.34706(3.6154)	
			Cluster-2735.7468(3.1837)	
	shikimate O-hydroxycinnamoyltransferase	EC:2.3.1.133	Cluster-2735.61020(2.6755)	
	coniferyl-alcohol glucosyltransferase	EC:2.4.1.111	Cluster-2735.39569(2.8803)	
			Cluster-2735.39572(3.3527)	
	caffeic acid 3-O-methyltransferase	EC:2.1.1.68	Cluster-2735.15861(4.595)	
			Cluster-2735.35895(3.5474)	
	caffeoyl-CoA O-methyltransferase	EC:2.1.1.104	Cluster-2735.33118(7.7083)	
			Cluster-2735.14045(8.3986)	
	coniferyl-aldehyde dehydrogenase	EC:1.2.1.68	Cluster-2735.46659(1.895)	
			Cluster-2735.46658(3.4776)	
			Cluster-2735.26219(5.8527)	
			Cluster-2735.40576(2.4474)	
	caffeoylshikimate esterase	EC:3.1.1.-		Cluster-2735.30970(-1.5252)
	ferulate-5-hydroxylase	F5H		Cluster-2735.14069(-3.2707)
				Cluster-2735.56444(-Inf)
	4-coumarate--CoA ligase	EC:6.2.1.12	Cluster-2735.28638(2.0704)	Cluster-2735.52148(-10.858)
				Cluster-2735.52147(-5.4595)
				Cluster-2735.56633(-4.5347)
				Cluster-2735.28184(-5.1444)
	cinnamoyl-CoA reductase	EC:1.2.1.44	Cluster-2735.16349(3.6263)	Cluster-2735.43590(-7.7896)
			Cluster-2735.3517(5.2749)	
			Cluster-2735.3518(5.0161)	
			Cluster-2735.3519(4.6567)	
			Cluster-2735.44862(Inf)	
			Cluster-2735.43589(4.2987)	
	beta-glucosidase	EC:3.2.1.21	Cluster-2735.48092(Inf)	Cluster-2735.12289(-3.4774)
			Cluster-2735.48710(Inf)	Cluster-2735.12285(-3.8595)
			Cluster-2735.48716(Inf)	Cluster-2735.48711(-5.1313)
			Cluster-2735.48709(4.5295)	Cluster-2735.16562(-2.0926)

		Cluster-2735.48708(4.6121)	Cluster-2735.16565(-1.9024)
		Cluster-2735.18467(4.3727)	Cluster-2735.7471(-3.3184)
		Cluster-2735.20117(4.6681)	Cluster-2735.7472(-3.0623)
		Cluster-2735.39063(3.3729)	Cluster-2884.0(-Inf)
		Cluster-2735.24053(3.4006)	Cluster-2735.54999(-3.3195)
		Cluster-7549.0(Inf)	Cluster-2735.46840(-3.8689)
			Cluster-2735.33766(-3.3962)
			Cluster-2735.19013(-3.5056)
			Cluster-2735.24889(-3.575)
			Cluster-2735.49092(-1.8759)
cinnamyl-alcohol dehydrogenase	EC:1.1.1.195	Cluster-2735.23731(2.2707)	Cluster-2735.53408(-11.06)
		Cluster-2735.35167(3.6415)	Cluster-2735.29307(-5.0014)
peroxidase	EC:1.11.1.7	Cluster-2735.49646(Inf)	Cluster-2735.22902(-4.0135)
		Cluster-3813.0(2.2003)	Cluster-2735.11953(-4.8292)
		Cluster-2735.36245(7.4857)	Cluster-2735.52377(-2.0749)
		Cluster-2735.28475(4.5868)	Cluster-2735.13100(-3.8592)
		Cluster-2735.30400(4.0282)	Cluster-2735.56922(-1.9681)
		Cluster-14289.2(Inf)	Cluster-2735.55038(-4.4426)
		Cluster-2735.15604(2.9501)	Cluster-2735.9670(-3.6483)
		Cluster-2735.38913(3.1316)	Cluster-2735.19010(-1.7626)
		Cluster-2735.31424(6.7848)	Cluster-2735.13099(-3.8453)
		Cluster-2735.27224(9.3383)	Cluster-2735.55356(-5.9941)
		Cluster-2735.34472(6.3871)	Cluster-2735.20112(-2.9545)
		Cluster-2735.32061(6.6137)	Cluster-7981.0(-3.9003)
		Cluster-2735.32060(5.8883)	Cluster-2735.13163(-9.2919)
		Cluster-2735.29950(3.307)	Cluster-2735.42980(-3.3465)
		Cluster-2735.29573(9.3576)	Cluster-2735.53738(-4.801)
		Cluster-2735.28697(2.3461)	Cluster-2735.52083(-3.1864)
		Cluster-2735.26177(Inf)	Cluster-2735.34943(-3.9301)
		Cluster-2735.22743(3.7294)	Cluster-2735.38427(-2.6209)
		Cluster-2735.38619(2.0294)	Cluster-2735.41274(-1.9998)
		Cluster-2735.37249(6.9364)	Cluster-2735.53836(-5.2696)
		Cluster-2735.44735(6.9387)	Cluster-2735.53835(-5.938)
		Cluster-2735.37494(Inf)	Cluster-2735.53064(-3.4249)
		Cluster-2735.15412(Inf)	Cluster-2735.39377(-3.1439)
		Cluster-2735.26780(Inf)	Cluster-2735.23099(-4.5331)
		Cluster-2735.42626(4.59)	Cluster-2735.51772(-7.3556)
			Cluster-2735.12776(-10.003)
coumaroylquinate 3'-monooxygenase	EC:1.14.1336	Cluster-2735.6467(3.8941)	Cluster-2735.35774(-1.8139)

Table S3. Results of the analysis of molecular variance (AMOVA) performed on six populations in *Pinus bungeana* using 17 microsatellite markers.

Source of variation	Sum of squares	Variance components	variation (%)	Fixation index
Among Populations	154.56	1.308	25.185	$F_{ST} = 0.252^{***}$
Among Individuals	474.042	3.886	74.815	
Total	628.602	5.194	100	

*P < 0.05, ** P < 0.01 and ***P < 0.001, 1000 permutations; F_{ST}, differentiation among populations within *Pinus bungeana*

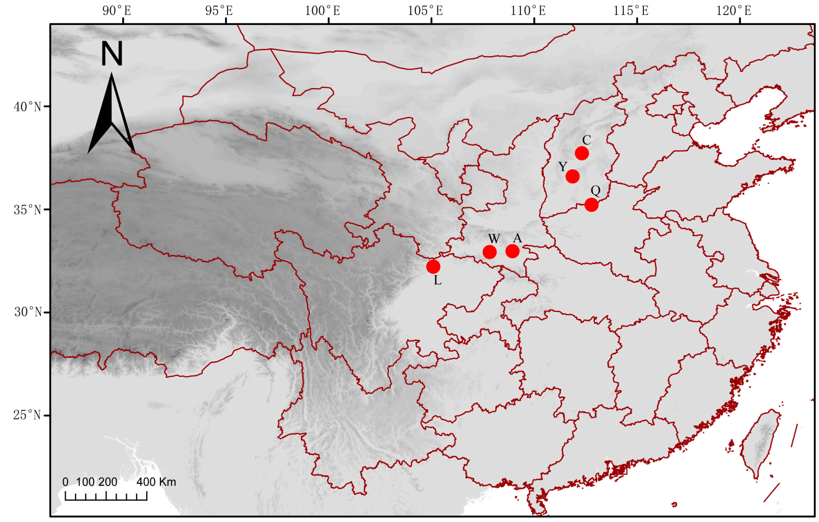
90°E
95°E
100°E
105°E
110°E
115°E
120°E
40°N
35°N
30°N
25°N
N
C
Y
Q
W
A
L
0 100 200 400 Km

Figure S1

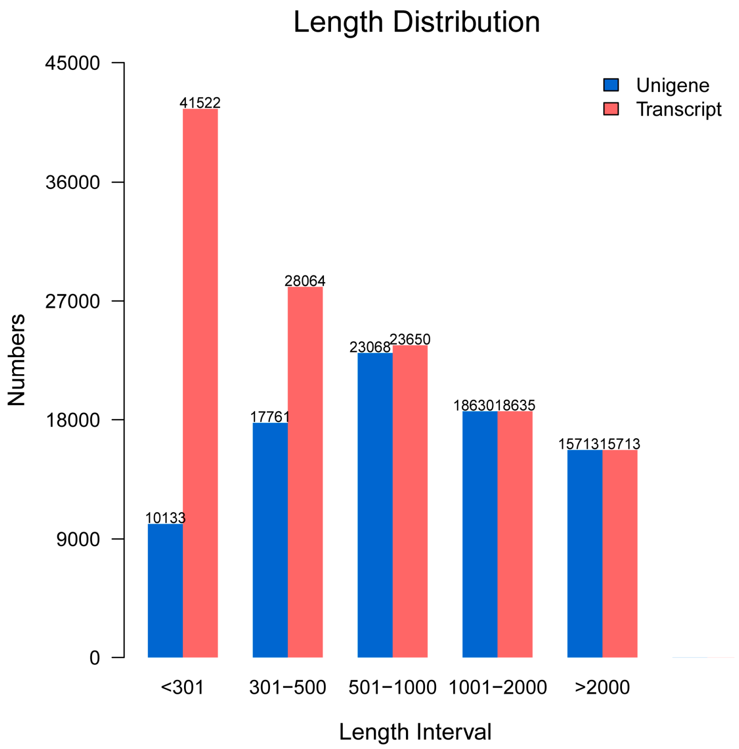
Length Distribution
45000
36000
27000
18000
9000
0
Numbers
Unigene
Transcript
41522
10133
28064
17761
23068
23650
18630
18635
15713
15713
<301
301−500
501−1000
1001−2000
>2000
Length Interval

Figure S2

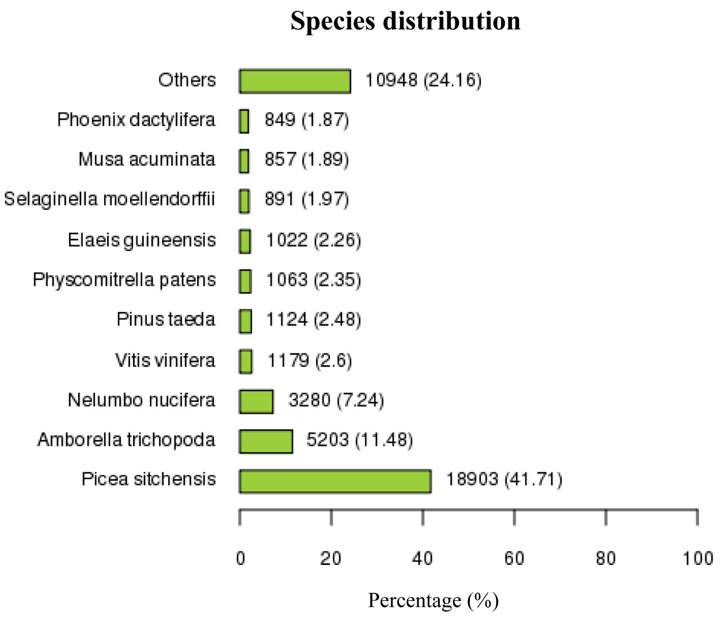

Species distribution
Others
10948 (24.16)
Phoenix dactylifera
849 (1.87)
Musa acuminata
857 (1.89)
Selaginella moellendorffii
891 (1.97)
Elaeis guineensis
1022 (2.26)
Physcomitrella patens
1063 (2.35)
Pinus taeda
1124 (2.48)
Vitis vinifera
1179 (2.6)
Nelumbo nucifera
3280 (7.24)
Amborella trichopoda
5203 (11.48)
Picea sitchensis
18903 (41.71)
0
20
40
60
80
100
Percentage (%)

Figure S3

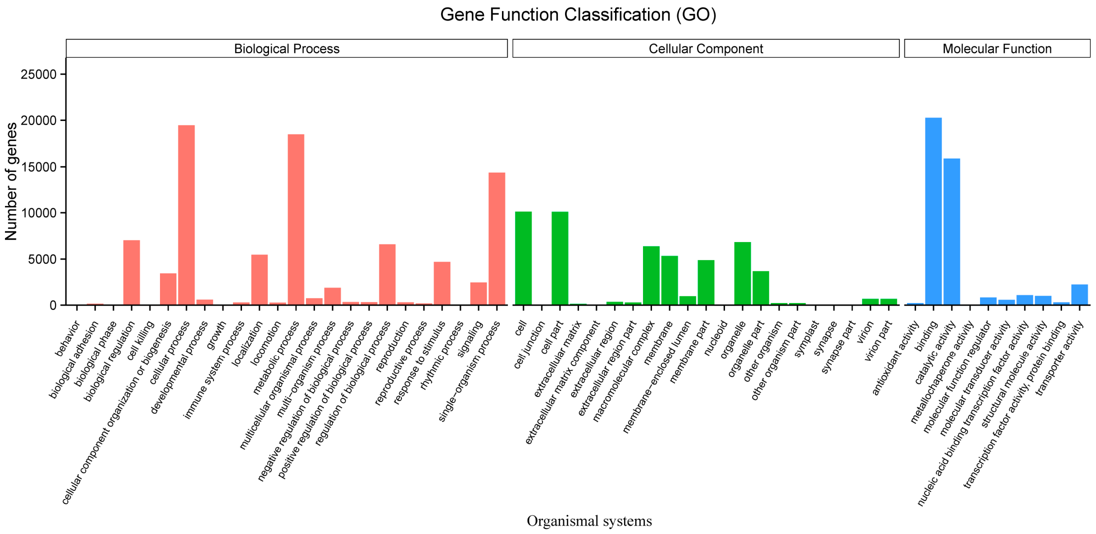
Gene Function Classification (GO)
Biological Process
Cellular Component
Molecular Function
Number of genes
25000
20000
15000
10000
5000
0
behavior
biological adhesion
biological phase
biological regulation
cell killing
cellular component organization or biogenesis
cellular process
developmental process
growth
immune system process
localization
locomotion
metabolic process
multicellular organismal process
multi−organism process
negative regulation of biological process
positive regulation of biological process
regulation of biological process
reproduction
reproductive process
response to stimulus
rhythmic process
signaling
single−organism process
cell
cell junction
cell part
extracellular matrix
extracellular matrix component
extracellular region
extracellular region part
macromolecular complex
membrane
membrane−enclosed lumen
membrane part
nucleoid
organelle
organelle part
other organism
other organism part
symplast
synapse
synapse part
virion
virion part
antioxidant activity
binding
catalytic activity
metallochaperone activity
molecular function regulator
molecular transducer activity
nucleic acid binding transcription factor activity
structural molecule activity
transcription factor activity, protein binding
transporter activity
Organismal systems

Figure S4

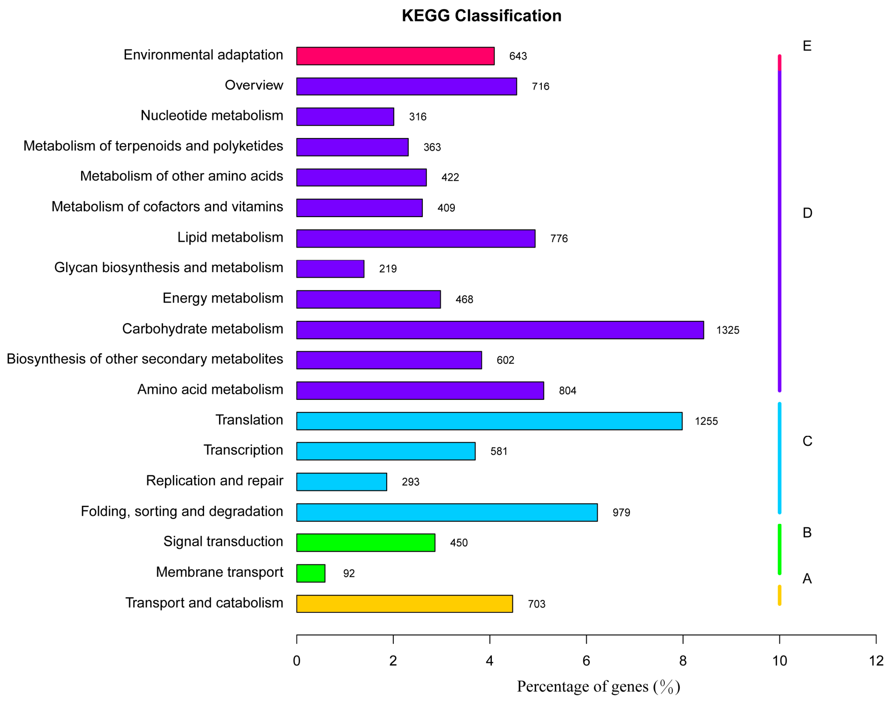
KEGG Classification
Environmental adaptation
643
Overview
716
Nucleotide metabolism
316
Metabolism of terpenoids and polyketides
363
Metabolism of other amino acids
422
Metabolism of cofactors and vitamins
409
Lipid metabolism
776
Glycan biosynthesis and metabolism
219
Energy metabolism
468
Carbohydrate metabolism
1325
Biosynthesis of other secondary metabolites
602
Amino acid metabolism
804
Translation
1255
Transcription
581
Replication and repair
293
Folding, sorting and degradation
979
Signal transduction
450
Membrane transport
92
Transport and catabolism
703
E
D
C
B
A
0
2
4
6
8
10
12
Percentage of genes (%)

Figure S5

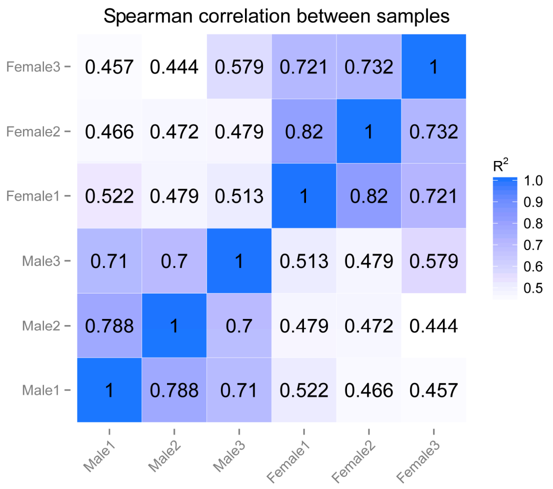

Spearman correlation between samples
Female3
Female2
Female1
Male3
Male2
Male1
0.457 0.444 0.579 0.721 0.732 1
0.466 0.472 0.479 0.82 1 0.732
0.522 0.479 0.513 1 0.82 0.721
0.71 0.7 1 0.513 0.479 0.579
0.788 1 0.7 0.479 0.472 0.444
1 0.788 0.71 0.522 0.466 0.457
Male1
Male2
Male3
Female1
Female2
Female3
R2
1.0
0.9
0.8
0.7
0.6
0.5

Figure S6

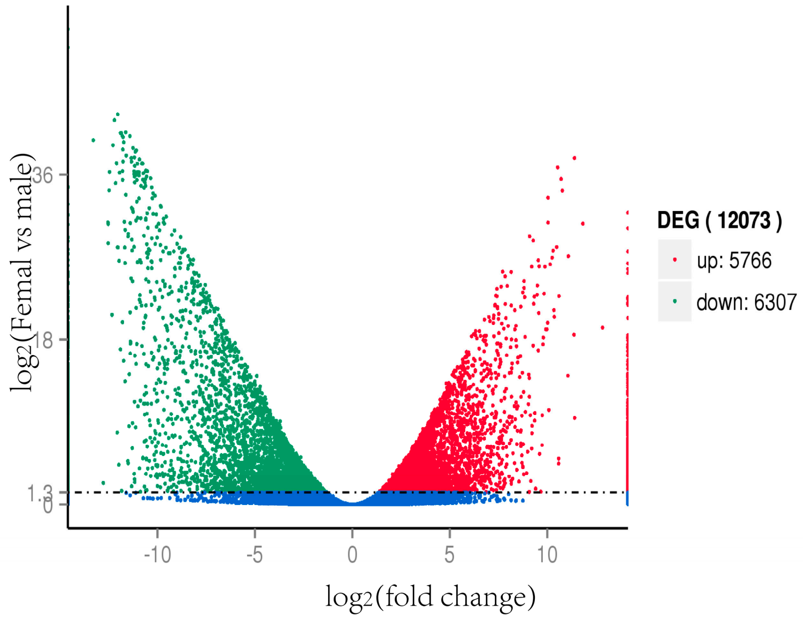
log2(Femal vs male)
36
18
1.3
0
-10
-5
0
5
10
log2(fold change)
DEG (12073)
up: 5766
down: 6307

Figure S7

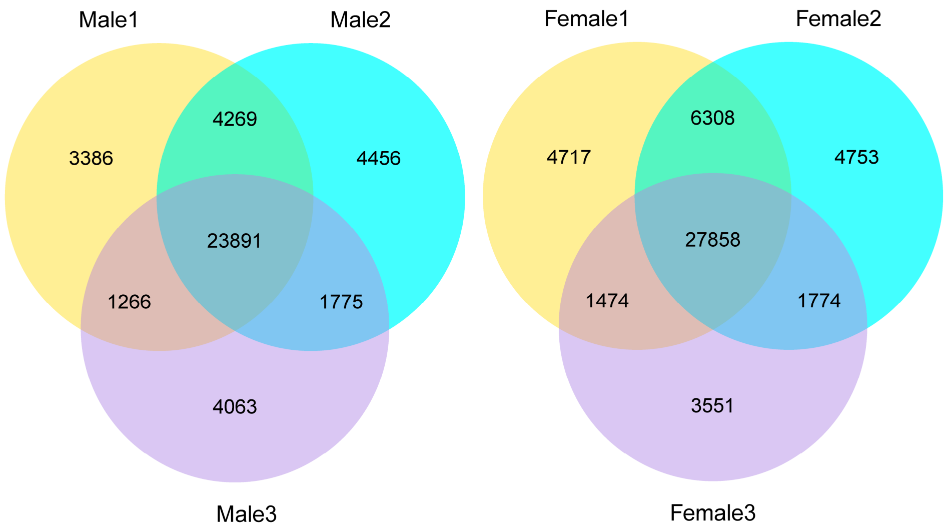
Male1
Male2
4269
3386
4456
23891
1266
1775
4063
Male3
Female1
Female2
6308
4717
4753
27858
1474
1774
3551
Female3

Figure S8

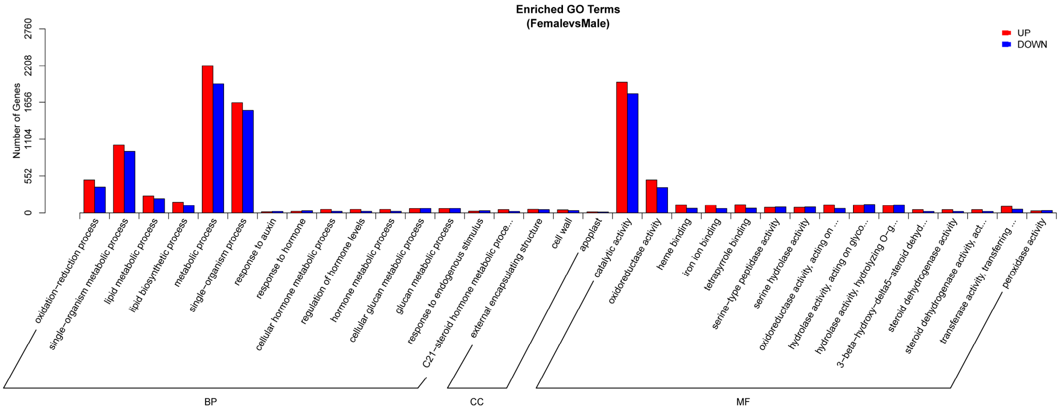

Enriched GO Terms
(FemalevsMale)
UP
DOWN
Number of Genes
0
552
1104
1656
2208
2760
oxidation−reduction process
single−organism metabolic process
lipid metabolic process
lipid biosynthetic process
metabolic process
single−organism process
response to auxin
response to hormone
cellular hormone metabolic process
regulation of hormone levels
hormone metabolic process
cellular glucan metabolic process
glucan metabolic process
response to endogenous stimulus
C21−steroid hormone metabolic proce...
external encapsulating structure
cell wall
apoplast
catalytic activity
oxidoreductase activity
heme binding
iron ion binding
tetrapyrrole binding
serine−type peptidase activity
serine hydrolase activity
oxidoreductase activity, acting on ...
hydrolase activity, acting on glyco...
hydrolase activity, hydrolyzing O−g...
3−beta−hydroxy−delta5−steroid dehyd...
steroid dehydrogenase activity
steroid dehydrogenase activity, act...
transferase activity, transferring ...
peroxidase activity
BP
CC
MF

Figure S9

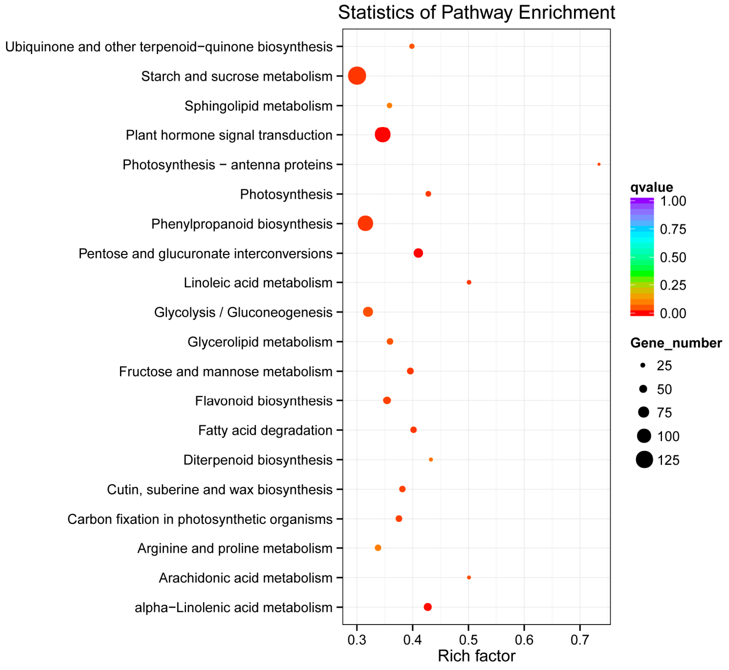

Statistics of Pathway Enrichment
Ubiquinone and other terpenoid−quinone biosynthesis
Starch and sucrose metabolism
Sphingolipid metabolism
Plant hormone signal transduction
Photosynthesis − antenna proteins
Photosynthesis
Phenylpropanoid biosynthesis
Pentose and glucuronate interconversions
Linoleic acid metabolism
Glycolysis / Gluconeogenesis
Glycerolipid metabolism
Fructose and mannose metabolism
Flavonoid biosynthesis
Fatty acid degradation
Diterpenoid biosynthesis
Cutin, suberine and wax biosynthesis
Carbon fixation in photosynthetic organisms
Arginine and proline metabolism
Arachidonic acid metabolism
alpha−Linolenic acid metabolism
0.3
0.4
0.5
0.6
0.7
Rich factor
qvalue
1.00
0.75
0.50
0.25
0.00
Gene_number
25
50
75
100
125

Figure S10

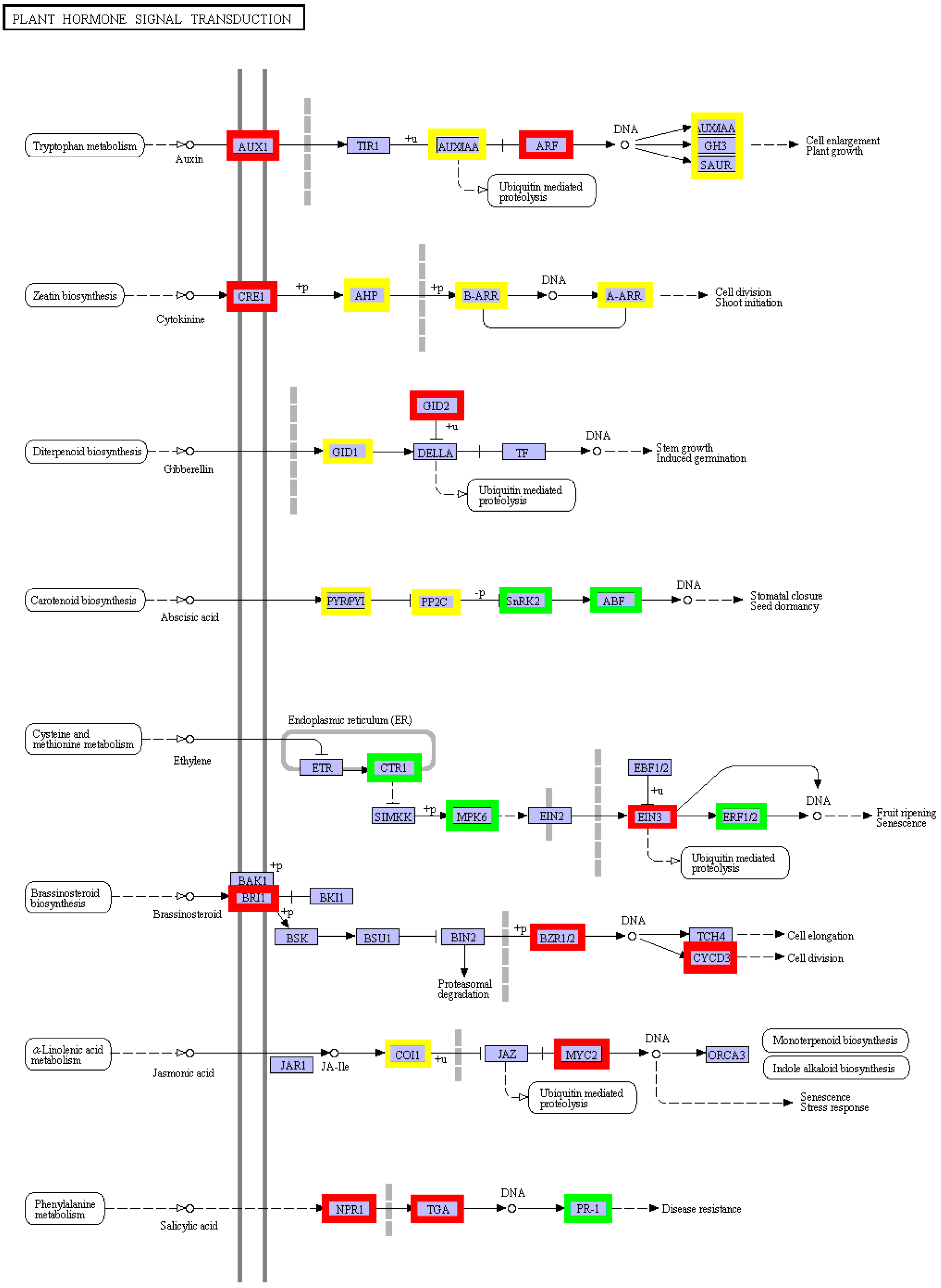

PLANT HORMONE SIGNAL TRANSDUCTION
Tryptophan metabolism
Auxin
AUX1
TIR1
+u
AUX/IAA
ARF
DNA
AUX/IAA
GH3
SAUR
Cell enlargement
Plant growth
Ubiquitin mediated proteolysis
Zeatin biosynthesis
Cytokinine
CRE1
+p
AHP
+p
B-ARR
DNA
A-ARR
Cell division
Shoot initiation
GID2
+u
Diterpenoid biosynthesis
Gibberellin
GID1
DELLA
TF
DNA
Stem growth
Induced germination
Ubiquitin mediated proteolysis
Carotenoid biosynthesis
Abscisic acid
PYR/PYL
PP2C
-p
SnRK2
ABF
DNA
Stomatal closure
Seed dormancy
Endoplasmic reticulum (ER)
Cysteine and methionine metabolism
Ethylene
ETR
CTR1
SIMKK
+p
MPK6
EIN2
EBF1/2
+u
EIN3
ERF1/2
DNA
Fruit ripening
Senescence
Ubiquitin mediated proteolysis
Brassinosteroid biosynthesis
Brassinosteroid
BAK1
+p
BRI1
BKI1
+p
BSK
BSU1
BIN2
Proteasomal degradation
+p
BZR1/2
DNA
TCH4
CYCD3
Cell elongation
Cell division
α-Linolenic acid metabolism
Jasmonic acid
JAR1
JA-Ile
COI1
+u
JAZ
MYC2
DNA
ORCA3
Monoterpenoid biosynthesis
Indole alkaloid biosynthesis
Ubiquitin mediated proteolysis
Senescence
Stress response
Phenylalanine metabolism
Salicylic acid
NPR1
TGA
DNA
PR-1
Disease resistance

Figure S11

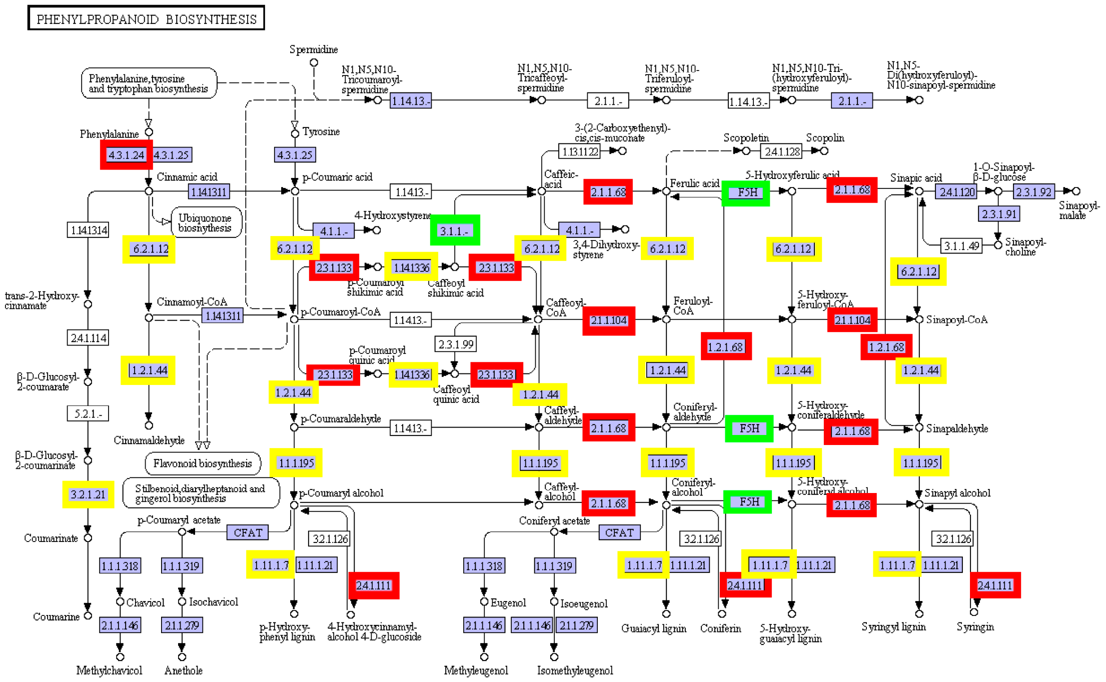
PHENYLPROPANOID BIOSYNTHESIS
Spermidine
Phenylalanine,tyrosine and tryptophan biosynthesis
Phenylalanine
Tyrosine
Cinnamic acid
p-Coumaric acid
Caffeic-acid
Ferulic acid
5-Hydroxyferulic acid
Sinapic acid
1-O-Sinapoyl-β-D-glucose
Sinapoyl-malate
Sinapoyl-choline
Ubiquonone biosynthesis
4-Hydroxystyrene
3,4-Dihydroxy-styrene
Scopoletin
Scopolin
trans-2-Hydroxy-cinnamate
Cinnamoyl-CoA
p-Coumaroyl-CoA
Caffeoyl-CoA
Feruloyl-CoA
5-Hydroxy-feruloyl-CoA
Sinapoyl-CoA
β-D-Glucosyl-2-coumarate
β-D-Glucosyl-2-coumarinate
Cinnamaldehyde
p-Coumaraldehyde
Caffeyl-aldehyde
Coniferyl-aldehyde
5-Hydroxy-coniferaldehyde
Sinapaldehyde
Flavonoid biosynthesis
Stilbenoid,diarylheptanoid and gingerol biosynthesis
p-Coumaryl alcohol
Caffeyl-alcohol
Coniferyl-alcohol
5-Hydroxy-coniferyl alcohol
Sinapyl alcohol
p-Coumaryl acetate
Coniferyl acetate
Coumarinate
Coumarine
Chavicol
Isochavicol
Eugenol
Isoeugenol
Methylchavicol
Anethole
Methyleugenol
Isomethyleugenol
p-Hydroxy-phenyl lignin
4-Hydroxycinnamyl-alcohol 4-D-glucoside
Guaiacyl lignin
Coniferin
5-Hydroxy-guaiacyl lignin
Syringyl lignin
Syringin

Figure S12

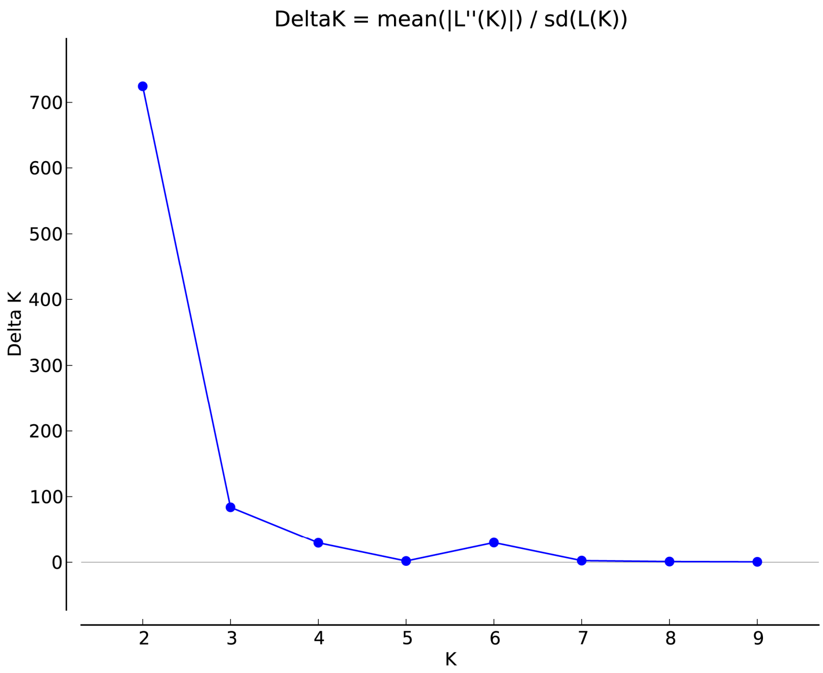
DeltaK = mean(|L''(K)|) / sd(L(K))
Delta K
700
600
500
400
300
200
100
0
2
3
4
5
6
7
8
9
K

Figure S13

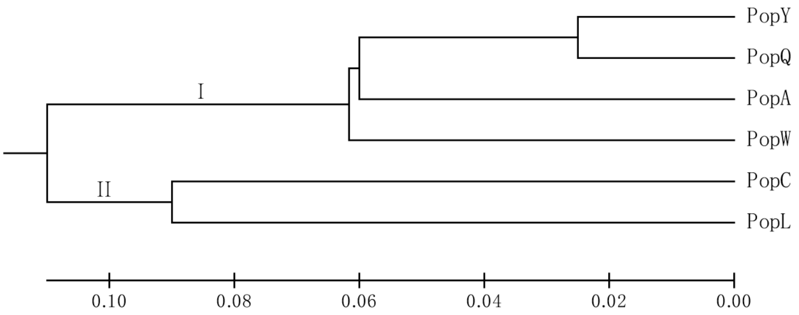

Figure S14
